# Health workforce demography: a framework to improve understanding of the health workforce and support achievement of the Sustainable Development Goals

**DOI:** 10.1186/s12960-020-0445-6

**Published:** 2020-01-29

**Authors:** Sylvia Szabo, Andrea Nove, Zoë Matthews, Ashish Bajracharya, Ibadat Dhillon, Devendra Raj Singh, Aurora Saares, James Campbell

**Affiliations:** 1 Asian Institute of Technology, 58 Moo 9, Km. 42, Paholyothin Highway, Klong Luang, Pathum Thani, 12120 Thailand; 2 Novametrics Ltd., 4 Cornhill Close, Duffield, Derbyshire DE56 4HQ UK; 3 Department of Social Statistics and Demography, University of Southampton, Highfield, Southampton, SO17 1BJ UK; 4 Population Council, Phnom Penh Center, Building B, 1st Floor, Rm 136, Street Sothearos, Khan Chamkar Morn, Phnom Penh, Cambodia; 5 World Health Organization, Avenue Appia 20, 1211 Geneva, Switzerland; 6 Asian College for Advanced Studies, Purbanchal University, Satdobatdo, Lalitpur, Kathmandu, Nepal

**Keywords:** Human resources for health, Health workforce planning, Health needs, Demand for health care, Nepal, Finland, Demography

## Abstract

The ambition of universal health coverage entails estimation of the number, type and distribution of health workers required to meet the population need for health services. The demography of the population, including anticipated or estimated changes, is a factor in determining the ‘universal’ needs for health and well-being. Demography is concerned with the size, breakdown, age and gender structure and dynamics of a population. The same science, and its robust methodologies, is equally applicable to the demography of the health workforce itself. For example, a large percentage of the workforce close to retirement will impact availability, a geographically mobile workforce has implications for health coverage, and gender distribution in occupations may have implications for workforce acceptability and equity of opportunity. In a world with an overall shortage of health workers, and the expectation of increasing need as a result of both population growth in the global south and population ageing in the global north, studying and understanding demographic characteristics of the workforce can help with future planning. This paper discusses the dimensions of health worker demography and considers how demographic tools and techniques can be applied to the analysis of the health labour market. A conceptual framework is introduced as a step towards the application of demographic principles and techniques to health workforce analysis and planning exercises as countries work towards universal health coverage, the reduction of inequities and national development targets. Some illustrative data from Nepal and Finland are shown to illustrate the potential of this framework as a simple and effective contribution to health workforce planning.

## Introduction

An adequate, well-distributed and motivated health workforce is central to the achievement of universal health coverage (UHC), and many of the Sustainable Development Goals (SDGs), including health (SDG3), decent work and economic growth (SDG8), gender equality (SDG5) and migration (SDG10) [1]. Achievement of SDG3 requires not only more health workers, but closer attention to and tailored policies that reflect health workforce characteristics and behaviour. This requires strengthened availability, quality, analysis and use of health workforce data to inform advocacy, planning, policy-making, governance and accountability at national, regional and global levels.

Despite the recent growth of the health sector, many countries have insufficient human resources for health (HRH). A supply-based shortage occurs when insufficient HRH are produced (or imported) and retained. A demand-based shortage occurs when a country cannot pay the recurrent costs of meeting the demand for HRH. In many low- and middle-income settings, both supply and economic demand fall short of population needs. It can be challenging to identify suitable methods for understanding the scale and characteristics of a shortage and therefore to devise appropriate policies for addressing it.

Demography is the study of the profile and habits of populations, e.g. their age, gender, ethnicity, fertility, mortality and migration patterns [2]. In this paper, we argue that understanding the demography of the workforce can help with workforce planning and management. Health worker demography is not a new concept: it was first discussed in the 1960s, when Bui-Ding-Ha-Doan suggested that the application of classic demographic analysis to sub-populations could be instructive [3]. He noted that people joining the health workforce can be likened to births, leavers can be likened to deaths and inflows and outflows can therefore be studied following similar principles to the analysis of births and deaths in the general population. He also pointed out that the general population supplies the sub-population of health workers, which exists to meet the health needs and demands of the general population. This interdependence brings with it a need to understand the demography of both populations.

While there are a few recent examples of using demographic techniques to study HRH [4], the concept of health worker demography as an important field of study has not yet gained traction. Workforce inflows and outflows are commonly included as parameters in planning models, especially in high-income countries [5]; however, it is rare for demographic tools and techniques to be applied when estimating and analysing the scale of these inflows and outflows. This paper aims to fill this gap and highlight the potential of health workforce demography as a field of study. It also aims to stimulate further use of demographic techniques and tools during workforce planning and the design of national- and local-scale development strategies. It focuses on the interpretation of population pyramids, but other tools and techniques are also applicable, e.g. the estimation of rates of entry to and exit from the workforce (especially where empirical data are sparse), population mapping (to understand the geographical distribution of the workforce) and survival analysis (to better understand the workforce attrition). This would contribute to a greater understanding of the health workforce and thus help to accelerate progress towards global and national goals.

## Impact of demography on the health workforce in different contexts

Demographic transition explains the decline from high to low rates of birth and death [6]. Countries can be classified as (1) pre-transition, with high birth and death rates; (2) early transition, when death rates start to decline but birth rates remain high, resulting in a rapid population growth; (3) late transition, when birth rates start to decline and population growth slows; and (4) post-transition, when both fertility and mortality rates are low and population growth is negligible or starts to decline. While many high-income countries experienced a transition in the past, many low- and middle-income countries (LMICs) are doing so now.

Countries in different stages of transition exhibit different patterns of health and health workforce needs and demand. LMICs in pre- and early transition experience high infant, child and maternal mortality [2] and have large cohorts of young people. Many pre- and early transition countries have critical shortages of health workers, especially in rural areas [7], often compounded by high levels of outmigration. The ability of countries to address population health has significant implications for economic and social development.

Countries in late transition or post-transition have ageing populations and high rates of chronic and noncommunicable diseases including diabetes and cancer. They require a health workforce with varied specialisms, skilled in the use of technology, and a high level of elderly care. An ageing population—and within it, an ageing workforce—brings with it concerns about the future supply of HRH and their capacity to perform physically demanding work [8]. The global needs-based shortage of health workers was estimated at almost 18 million in 2013, being the largest in South East Asia and Africa, and is projected to be 15 million by 2030 and to worsen in low-income countries [9].

A commonly used and easily understood demographic analysis tool is the population pyramid, which is a graphical representation of the age and sex composition of a population. Pyramids can help to generate hypotheses about something that changed in the past (e.g. phenomena such as war, epidemics, mass migration, sex-selective abortion and natural disasters can cause ‘dents’ to appear in one or both sides of a pyramid). They can also contribute to forecasts of how population size and structure might change in the future, e.g. a broad base predicts population growth and a narrow base indicates contraction. This predictive ability can be useful for future planning.

**Fig. 1:**
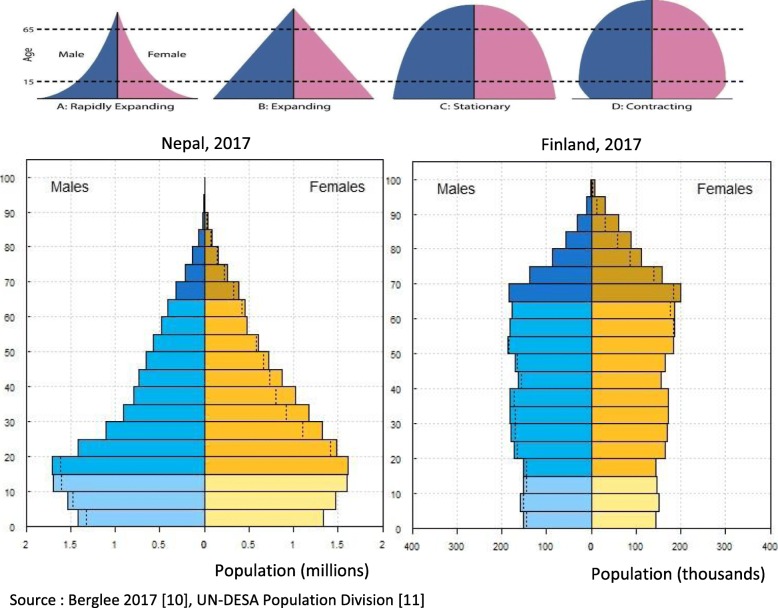
Population pyramids. Source: Berglee [10] and UN-DESA Population Division [11]

Figure 1 shows the four basic shapes that are commonly observed in general populations: (1) a wide-based pyramid with a narrow top, illustrating a fast-growing population with high fertility and low life expectancy; (2) a classic pyramid shape, which illustrates an expanding population due to high fertility and slightly better life expectancy; (3) a stationary population, with births and deaths being fairly evenly balanced; and (4) a contracting population, with low fertility and long life expectancy. Figure 1 also shows the population pyramids for Nepal and Finland, countries that are in different stages of their demographic transition and with contrasting population profiles, and therefore different implications for HRH. Nepal’s pyramid shows a population that until recently was expanding but is now starting to contract. There are gender imbalances, with an excess of female adults and male children. Finland’s pyramid shows a fairly stationary, but slightly contracting, population with a fairly equal balance between the sexes. These population pyramids also constitute useful reference points when analysing the HRH pyramids (presented later in this paper).

The principles of population demography can be applied to the health workforce. The health workforce is a sub-population within the general population, and parallels can be drawn between key demographic events in these two populations. For example, the rate of production of HRH is to the health worker population what the fertility rate is to the general population. Similarly, the rate at which HRH leave the workforce permanently is like the rate at which people die in the general population, and the rate at which health workers join or leave the population temporarily is like the rate of migration in the general population. Age, gender and ethnic or geographic imbalances in the health workforce can bring about challenges in the same way that such imbalances in the general population do.

## A conceptual framework for health worker demography

**Fig. 2:**
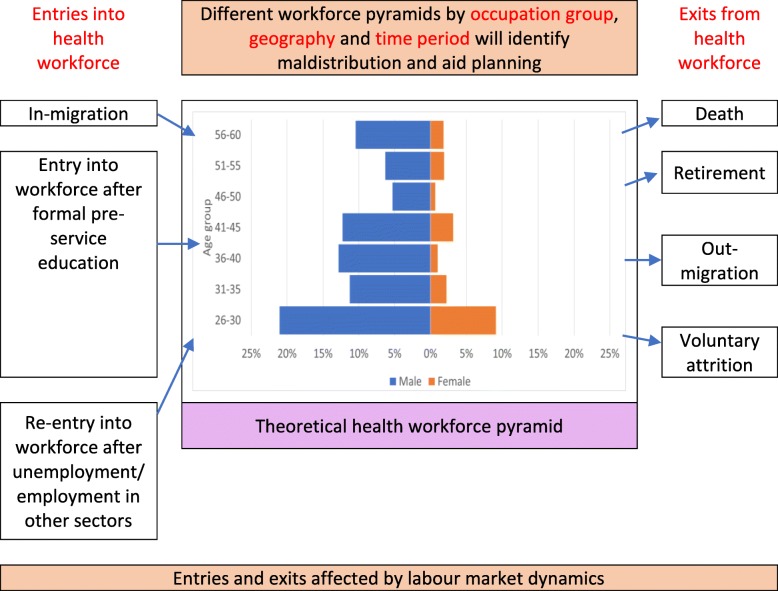
Conceptual framework of health workforce demography

Figure 2 presents a conceptual framework for health workforce demography which focuses on key types of entry and exit, the nature of which can influence the age and gender profile of the workforce, as illustrated by the (fictional) pyramid in the centre of the diagram. The main entry elements relate to education, in-migration and entry/re-entry into the workforce after a temporary break. These factors tend to be inter-related and vary across different geographical contexts, regulatory systems and occupations (which can also be highly gendered). Exit elements include out-migration, lack of retention (e.g. after family formation) and involuntary exits (e.g. dismissal, long-term illness, retirement, death). These elements can vary by age, gender and occupation group, which makes it vital to collect and analyse high-quality HRH data, disaggregated by these variables [11].

Demographic trends can have direct implications for labour markets through three primary channels: labour supply, productivity and demand [12]. While labour supply and demand are more intuitive, worker productivity may also be affected as for example ageing populations may be arguably at risk of lower innovative capacity and productive outputs [12]. Relevant policy areas include health worker production, distribution, efficiency, migration, regulation and the role of the private sector. The education of health workers is a critical factor affecting the supply and gender balance of the health workforce.

## Example analyses: physicians and nurses in Nepal and Finland

In this section, HRH pyramids for physicians, nurses and midwives in Nepal and Finland are used to illustrate contrasting health workforce demographics. The data sources for the two countries use different age groupings, so a direct comparison is not possible, however, then can be easily compared against the national pyramids (Fig. 1). Also, the gender and age patterns can be observed across the HRH across both populations.

Creating population pyramids requires robust data for each health worker category (e.g. physicians, nurses, midwives), their gender and their age/age group. HRH disaggregation should also take into account disaggregation by sector, employment patterns (e.g. full time vs part time) and geographical distribution. In this study, the Nepal data were derived from the Ministry of Health’s Human Resource Development Information System (HuRDIS). HuRDIS is a digital record of public sector HRH distribution. A 2012 HRH assessment identified over 50 000 health workers of whom 46% were female [13]. The total identified from HuRDIS across all occupation groups was just under 20 000 of which 39% were female. The data used for this analysis were triangulated with records at the Department of Health Services, but because of the limitations described above, the analysis should be regarded as illustrative only: an example of the potential of demographic techniques for workforce analysis and planning.

The Finland data are derived from Statistics Finland’s open database of employment statistics [14] and describe the occupation of employed permanent residents. The occupation groups are classified according to the Classification of Occupations 2010. The occupations included in this paper are physicians, nurses and midwives [15]. Occupation data are primarily based on a person’s main employment contract, so may include employees on parental or other types of temporary leave. Also, people employed though unemployment services (placements, training etc.) are counted.

**Fig. 3:**
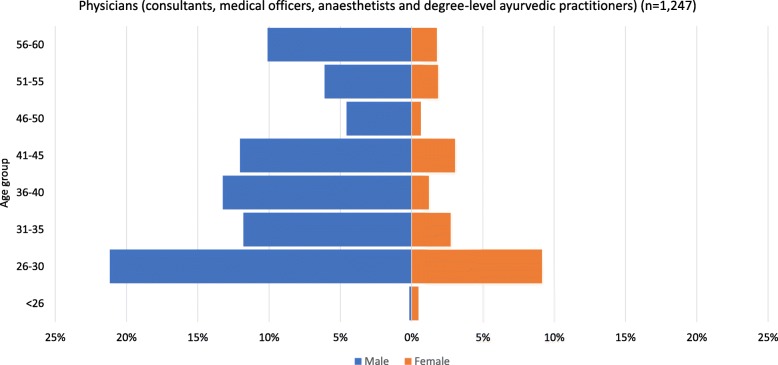
Age and gender profile of physicians deployed under Nepal’s national health services system, 2017. Source: derived from Nepal Ministry of Health, Human Resource Development Information System (HuRDIS) December 2017

**Fig. 4:**
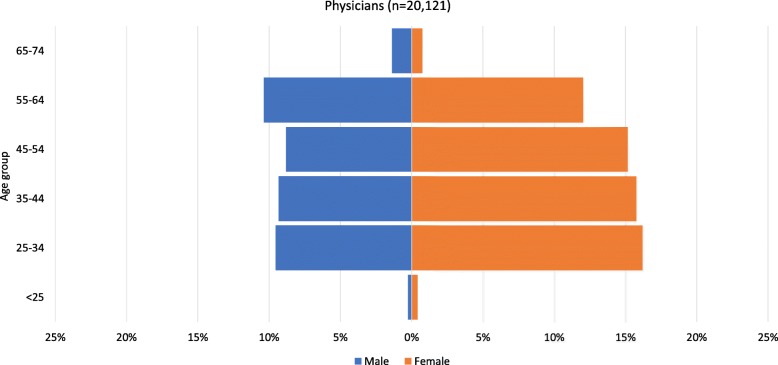
Age and gender profile of Finland’s physicians with a current contract of employment, 2015. Source: Statistics Finland [14]

Using population pyramids, Figs. 3 and 4 illustrate the age and gender profile of physicians in the datasets from the two countries. In Nepal, this includes both allopathic (*n* = 1200) and ayurvedic (*n* = 47) physicians. Allopathic physicians include medical officers, consultants and anaesthetists. Ayurvedic physicians include those educated to a degree level (a 5.5-year Bachelor of Ayurvedic Medicine and Surgery programme). In Finland, this includes all physicians with a current contract of employment (*n* = 20 121).

Several patterns may be observed in Figs. 3 and 4. First, there are gender imbalances, with many more male physicians than female in all age groups in Nepal (21% are female). This number is similar to that reported in the 2012 HRH assessment, which estimated that 25% of public sector physicians were female [13]. However, the relatively wide base of the pyramid on the female side indicates that the trend may be starting to change. Smaller numbers of women in the older age groups might partly date from the time when there was little or no medical education available in Nepal, so study abroad was the only way to qualify. By contrast, in Finland, women represent 60% of the physicians and are more numerous in all age groups except the oldest.

Second, the overall shape of the Finland pyramid indicates a stationary population which is unlikely to change significantly in size in the foreseeable future. By contrast, the Nepal pyramid has a wide base, predicting growth (assuming young physicians remain in the workforce), perhaps due to the recent expansion in capacity for medical education [16, 17]. However, on the male side, the pyramid has a broad top, which means that large numbers are likely to retire in the next few years, which will, in the short term, counterbalance growth due to large numbers of new graduates. It should, however, be noted that the wide top of the pyramid may be at least partly due to recent retirements not being recorded.

Third, there are small ‘dents’ in the Nepal pyramid: there are relatively few men aged 31–35 and 46–55 and relatively few women in their 30s. It is of course possible that these are due to the incomplete dataset. However, if a country shows such patterns, it is possible to speculate about possible reasons. For example, in Nepal, younger physicians tend to be more attracted to the private sector, which accounts for approximately 40% of health workers [13], which would result in the public sector workforce lacking younger physicians. Similarly, recruitment freezes (e.g. due to conflicts or disasters) may result in ‘missing’ cohorts in particular age groups. Dents in the female side may indicate low retention of women in their childbearing years and a need for policies to make it easier to combine a medical career with motherhood.

**Fig. 5:**
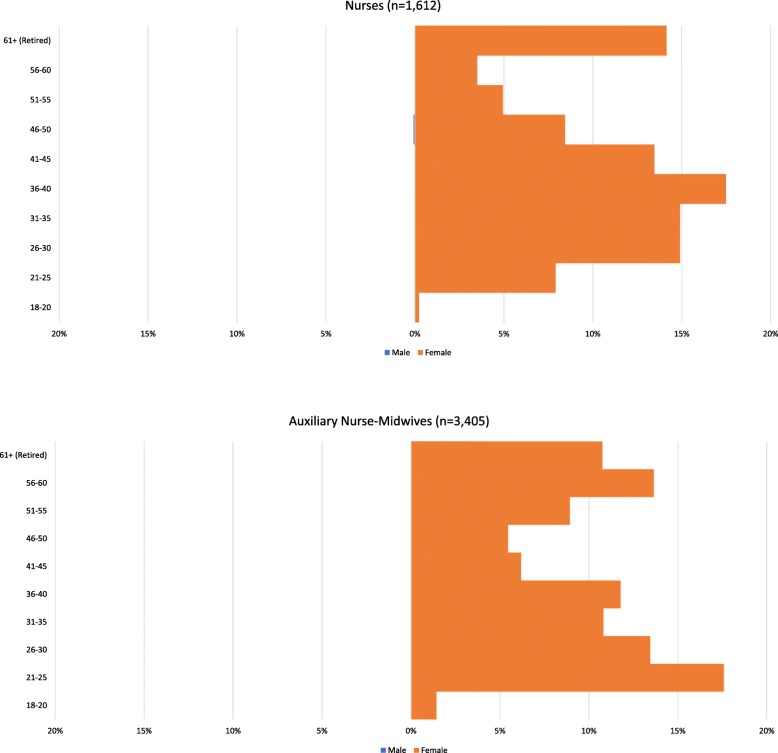
Age and gender profile of nurses and auxiliary nurse-midwives deployed under Nepal’s national health services system, 2017. Source: derived from Nepal Ministry of Health, Human Resource Development Information System (HuRDIS) December 2017

**Fig. 6:**
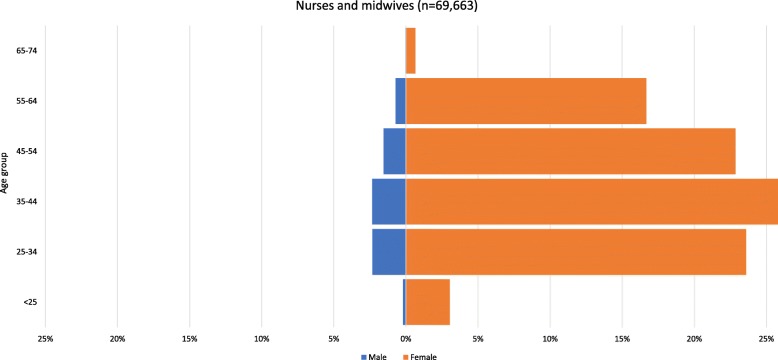
Age and gender profile of Finland’s nurses and midwives with a current contract of employment, 2015. Source: Statistics Finland [14]

Figures 5 and 6 illustrate the age and gender profile of Nepal’s nurses and auxiliary nurse-midwives (ANMs) and Finland’s nurses and midwives. The Nepal nurses graph includes staff nurses (3 years of nursing education) and graduate nurses (4–6 years of nursing education). ANMs study for 18 months to specialise in midwifery, reproductive health and community health in rural areas.

Observations can again be made, most notably the gender imbalance, with these occupations consisting mostly or completely of women. The lack of men in Nepal reflects the fact that only women can be ANMs and that, although men may train as nurses, they tend not to. As of 2018, official policy is that 15% of nursing education places are reserved for men [18].

Second, the relatively broad bases of the Nepal pyramids indicate large numbers of young nurses and ANMs joining the workforce and setting it up for growth if they are retained. However, fairly large numbers of ANMs are set to retire in the next few years, which will offset the growth in the short term. The largest age group among nurses and midwives in Finland is 35–44, and the shape of the pyramid indicates a fairly stable population.

Third, the Nepal nurses pyramid shows a large dent in the 31–35 age group, which again may be due to the incomplete database. However, it may be due to childbearing, out-migration, loss to the private sector or a combination of these things and should therefore be considered by workforce planners and policy-makers. Evidence suggests that Nepal educates more nurses than it employs [19], indicating that out-migration (including training for export) is significant, perhaps due to dissatisfaction with remuneration and career opportunities. There is no similar dent in the pyramid of Finnish nurses and midwives, which suggests that there are policies and laws regulating social protection and promoting female labour market participation, including parental leave, child benefit, childcare support, leave entitlements to take care of sick family members and employment security after parental leave [20].

## Key health worker demography issues: gender, migration and ageing

Some demographic issues cut across all or most elements of the conceptual framework proposed above, most notably gender, migration and ageing. These cross-cutting issues are discussed below and should be taken into account in any workforce analysis and planning exercise.

A greater awareness of gender in the study of the health workforce would help to address equity of access to health services and improve equity of opportunity within the workforce [21]. Where gender-disaggregated data are available, they tend to show that health occupations are highly gendered. Traditionally, most nursing, midwifery, community healthcare workers and long-term care workers are women, and most senior physicians and managers are men. However, gender and age data are not always easily available from HRH information systems [22], which is a barrier to evidence-based understanding of gender issues. Information on and action towards gender-transformative approaches and policies to overcome gender biases and inequalities in education and the health labour market are fundamentally important to the health sector and SDGs 5 and 8. The *Global Health Workforce Network* (established in 2017) includes a *Data and Evidence Hub* and a *Gender Equity Hub*, bringing together experts in strengthening data and evidence and supporting gender-transformative research, actions and investments [23].

Those working in female-dominated health occupations have often struggled to be recognised as skilled, autonomous professionals, and those working in long-term care roles have struggled to be recognised as health workers at all [22, 24]. The lack of professional recognition is a disincentive for well-qualified youth to consider a health worker career and is a barrier to career progression [22]. There is also evidence that a gender imbalance in health workforce leadership can constrain the health agenda and be a barrier to achieving health goals. Personal safety is a gendered issue, with female workers being more likely than male ones to experience violence and sexual harassment at work [25]. If suitable arrangements for parental leave, flexible working and childcare are not in place, female health workers may find it difficult to continue working after childbearing [26, 27], to the detriment of gender equity and workforce retention. Similarly, cultural expectations that women should care for elderly relatives may disproportionately affect the retention of female health workers [28, 29].

Thus, a lack of attention to gender issues may lead to significant—yet poorly understood—losses to the profession both in terms of entries (if talented youth choose alternative professions) and exits (if competent workers are not motivated to remain in the health workforce or return to it after a period of absence). Gender stereotypes are a constraint to men entering female-dominated professions and to women entering male-dominated professions, thus reducing the pool of potential recruits [34]. In a world with a shortage of health workers, such losses and constraints represent a significant waste of resources.

Given the feminisation of the health workforce, investing increasing resources in HRH can potentially catalyse women’s empowerment and equity and address the gender issues above. To realise these gains, we must first understand the context-specific composition of the health worker population at both national and sub-national levels, using demographic analysis techniques such as those described in this paper.

The international mobility of health workers is increasing [30]. Future projections point to a continuing acceleration in international migration [31]. Implementation of the WHO *Global Code of Practice* [32] shows a pattern of movement that is more complex than generally perceived, with a blurring of traditional ‘source’ and ‘destination’ countries. Temporary employment and recognition in multiple jurisdictions is also becoming increasingly common, e.g. only 20% of those who qualified in South Africa and registered in Ireland reported ‘only working in Ireland’.

A third cross-cutting issue is the ageing of the health workforce. As in many countries, the general population is ageing, so too is the sub-population of health workers. Understanding of the demographics and age distribution of the health workforce is needed to plan future needs, supply and demand, taking into consideration health workforce ageing and retirement. Where empirical data is sparse, demographic methods for estimating entries and exits to a population could be a useful addition to the toolkit. As health workers approach retirement, adequate numbers of new HRH need to be educated, recruited and retained, and early retirements minimised by reinvesting in mature health workers via supportive policies and practices.

## Conclusions

The study of populations is crucial for health planning. While the existing literature provides evidence regarding populations, their health status and changing health needs and demands, the scientific response to examining the demography of health workers has not yet caught up with the need to understand this evolving and diverse sub-population. This paper aims to contribute to furthering this understanding by suggesting a conceptual framework for studying health worker demography, using data from Nepal and Finland to illustrate the potential of this approach. It complements the existing literature on the socioeconomic impacts of investments in HRH and underlines the need to link these studies with demographic assessments of health worker populations.

Simple demographic techniques such as population pyramids do not require extensive statistical expertise but can enhance understanding of the health workforce and thus help examine what policy issues need to be addressed. For example, a drop in numbers among women in their early 30s may suggest a need for policies to address the retention of women with young families. These techniques can also help with medium-term forecasting without the need for complex models, because the shape of the pyramid predicts growth or contraction in workforce size and gives pointers on important issues such as outflows from the workforce in specific age and gender groups. However, these techniques are dependent on reliable data, which are not always easily available.

Due to data limitations, our sample pyramids focused on public sector only and did not account for informal workers providing home care. Also, local appointments and graduates on government bonded service are not generally included. Further studies including this disaggregation as well as looking at issues of formally retired (but still active) HRH might be useful to shed further light on the dynamics of health worker demography.

Consolidating studies on health worker migration, ageing and gender would also stimulate a critical policy agenda. Policy change on HRH has far-reaching and long-term effects on the health sector, population health and sustainable development. Information on health workforce demography is a cornerstone of health planning and policy, enabling more efficient and effective delivery of health interventions to populations, as well as to global priorities related to the creation of jobs, gender equality and international migration. Using simple analytical approaches, like the one presented in this study, would help public health professionals and development planners to formulate more effective evidence-based strategies as well as creating specific planning tools to implement policies.

## Data Availability

The datasets used and/or analysed during the current study are available from the corresponding author on reasonable request.
